# Plasma growth hormone is a potential biomarker of response to atezolizumab and bevacizumab in advanced hepatocellular carcinoma patients

**DOI:** 10.18632/oncotarget.28322

**Published:** 2022-12-06

**Authors:** Yehia I. Mohamed, Dan G. Duda, Muhammad O. Awiwi, Sunyoung S. Lee, Lina Altameemi, Lianchun Xiao, Jeffrey S. Morris, Robert A. Wolff, Khaled M. Elsayes, Rikita I. Hatia, Aliya Qayyum, Shadi M. Chamseddine, Asif Rashid, James C. Yao, Armeen Mahvash, Manal M. Hassan, Hesham M. Amin, Ahmed Omar Kaseb

**Affiliations:** ^1^Department of Gastrointestinal Medical Oncology, The University of Texas MD Anderson Cancer Center, Houston, TX 77030, USA; ^2^Steele Laboratories, Department of Radiation Oncology, Massachusetts General Hospital and Harvard Medical School, Boston, MA 02114, USA; ^3^Department of Diagnostic Imaging, The University of Texas MD Anderson Cancer Center, Houston, TX 77030, USA; ^4^Hurley Medical Center, Michigan State University, East Lansing, MI 48824, USA; ^5^Department of Biostatistics, The University of Texas MD Anderson Cancer Center, Houston, TX 77030, USA; ^6^Department of Biostatistics, Epidemiology, and Biostatistics, University of Pennsylvania Perelman School of Medicine, Philadelphia, PA 19104, USA; ^7^Department of Epidemiology, The University of Texas MD Anderson Cancer Center, Houston, TX 77030, USA; ^8^Department of Pathology, The University of Texas MD Anderson Cancer Center, Houston, TX 77030, USA; ^9^Department of Interventional Radiology, Division of Diagnostic Imaging, MD Anderson Cancer Center, Houston, TX 77030, USA; ^10^Department of Hematopathology, The University of Texas MD Anderson Cancer Center, Houston, TX 77030, USA

**Keywords:** growth hormone, hepatocellular carcinoma, immunotherapy, atezolizumab, bevacizumab

## Abstract

Introduction: Hepatocellular carcinoma (HCC) has limited systemic therapy options when discovered at an advanced stage. Thus, there is a need for accessible and minimally invasive biomarkers of response to guide the selection of patients for treatment. This study investigated the biomarker value of plasma growth hormone (GH) level as a potential biomarker to predict outcome in unresectable HCC patients treated with current standard therapy, atezolizumab plus bevacizumab (Atezo/Bev).

Materials and Methods: Study included unresectable HCC patients scheduled to receive Atezo/Bev. Patients were followed to determine progression-free survival (PFS) and overall survival (OS). Plasma GH levels were measured by ELISA and used to stratify the HCC patients into GH-high and GH-low groups (the cutoff normal GH levels in women and men are ≤3.7 μg/L and ≤0.9 μg/L, respectively). Kaplan-Meier method was used to calculate median OS and PFS and Log rank test was used to compare survival outcomes between GH-high and -low groups.

Results: Thirty-seven patients were included in this analysis, of whom 31 were males and 6 females, with a median age of 67 years (range: 37–80). At the time of the analysis, the one-year survival rate was 70% (95% CI: 0.51, 0.96) among GH low patients and 33% (95% CI: 0.16, 0.67) among GH high patients. OS was significantly superior in GH-low compared to GH-high patients (median OS: 18.9 vs. 9.3 months; *p* = 0.014). PFS showed a non-significant trend in favor of GH-low patients compared to the GH-high group (median PFS: 6.6 vs. 2.9 months; *p* = 0.053).

Discussion and conclusions: Plasma GH is a biomarker candidate for predicting treatment outcomes in advanced HCC patients treated with Atezo/Bev. This finding should be further validated in larger randomized clinical trials in advanced HCC patients.

## INTRODUCTION

Hepatocellular carcinoma (HCC) is one of the most common cancers worldwide and the fourth leading cause of cancer related mortality [1], with more than 80% of patients diagnosed at an advanced unresectable stage and curative options such as surgical resection and liver transplantation are excluded at these advanced stages, and therefore only limited effective treatment options remain [2]. Sorafenib and recently lenvatinib, have been approved as first line systemic treatment options for advanced HCC patients, as evidenced by several studies demonstrating the survival benefit of sorafenib and lenvantinib treatment [3]. The SHARP trial has demonstrated the survival benefit of sorafenib compared to placebo. In addition, lenvantinib was proven to be non-inferior to sorafenib in term of OS in the REFLECT trial [3, 4].

Outcomes of the IMbrave 150 trial changed the landscape for unresectable HCC treatment by showing superior overall survival (OS) and progression-free survival (PFS) in HCC patients receiving atezolizumab and bevacizumab (Atezo/Bev) compared to sorafenib with an acceptable adverse event profile [5, 6]. Hence, Atezo/Bev has become the first-line treatment option for patients with unresectable HCC.

GHR has been associated with different malignancies and disease progression (including breast cancer and HCC) [7–10]. Notably, the important role of growth hormone (GH)/growth hormone receptor (GHR) signaling in HCC development and tumor burden has been recently described by our group [7].

Many studies have demonstrated the efficacy and safety of Atezo/Bev in advanced HCC. However, there is a significant research gap in regard the biomarker value of plasma GH level in advanced HCC patients treated with atezolizumab plus bevacizumab. The present study was designed to investigate the association between GH levels and overall survivals (OS) and progression free survival (PFS) in HCC patients treated with current standard, atezolizumab plus bevacizumab.

## RESULTS

The study included 37 patients with advanced HCC who received atezolizumab plus bevacizumab at MDACC between June 2018 to November 2021 of whom 45.9% were 61–70 years of age at the time of diagnosis with a median age of 67 years (range: 37, 80), and 83.8% were males.

The characteristics of these patients were documented at the time of atezolizumab plus bevacizumab administration with 81% of patients had an Eastern Cooperative Oncology Group Performance Status (ECOG PS) score of 0, and 19% had a score of 1. Most of these patients had Child–Pugh classification A (78.4%), and only 8 (21.6%) patients had Child–Pugh classification B. The demographic characteristics of the patients are presented in Table 1.

**Table 1:** Baseline clinical characteristics of patients with hepatocellular carcinoma treated with atezolizumab plus bevacizumab

Variable	Number of patients (%)
**Age, years**	
≤ 40	1 (2.7)
41–50	1 (2.7)
51–60	5 (13.5)
61–70	17 (45.9)
> 70	13 (35.1)
**Diabetes**	
No	27 (72.9)
Yes	10 (27.1)
**Sex**	
Female	6 (16.2%)
Male	31 (83.8%)
**History of drinking alcohol**	
No	26 (70.2)
Yes	11 (29.7)
**Family history of liver cancer**	
No	30 (81)
Yes	6 (16.2)
Unknown	1 (2.7)
**Personal History of non-HCC cancer**	
No	27 (72.9)
Yes	10 (27.1)
**Race**	
Asian	4 (10.8)
Black	5 (13.5)
White	25 (67.5)
Unknown	3 (8.1)
**History of tobacco use**	
No	17 (45.9)
Yes	20 (54)
**Ascites**	
None	28 (75.6)
Slight	9 (24.3)
**Evidence of cirrhosis**	
No	8 (21.6)
Yes	28 (75.6)
Unknown	1 (2.7)
**ECOG performance status**	
0	30 (81)
1	7 (19)
**CTP class**	
A	29 (78.4%)
B	8 (21.6%)
**Metastasis**	
No	24 (64.9%)
Yes	13 (35.1%)

Abbreviations: CTP: Child-Turcotte-Pugh; ECOG: Eastern Cooperative Oncology Group; HCC: Hepatocellular carcinoma.

### Overall survival (OS)

**Figure 1:**
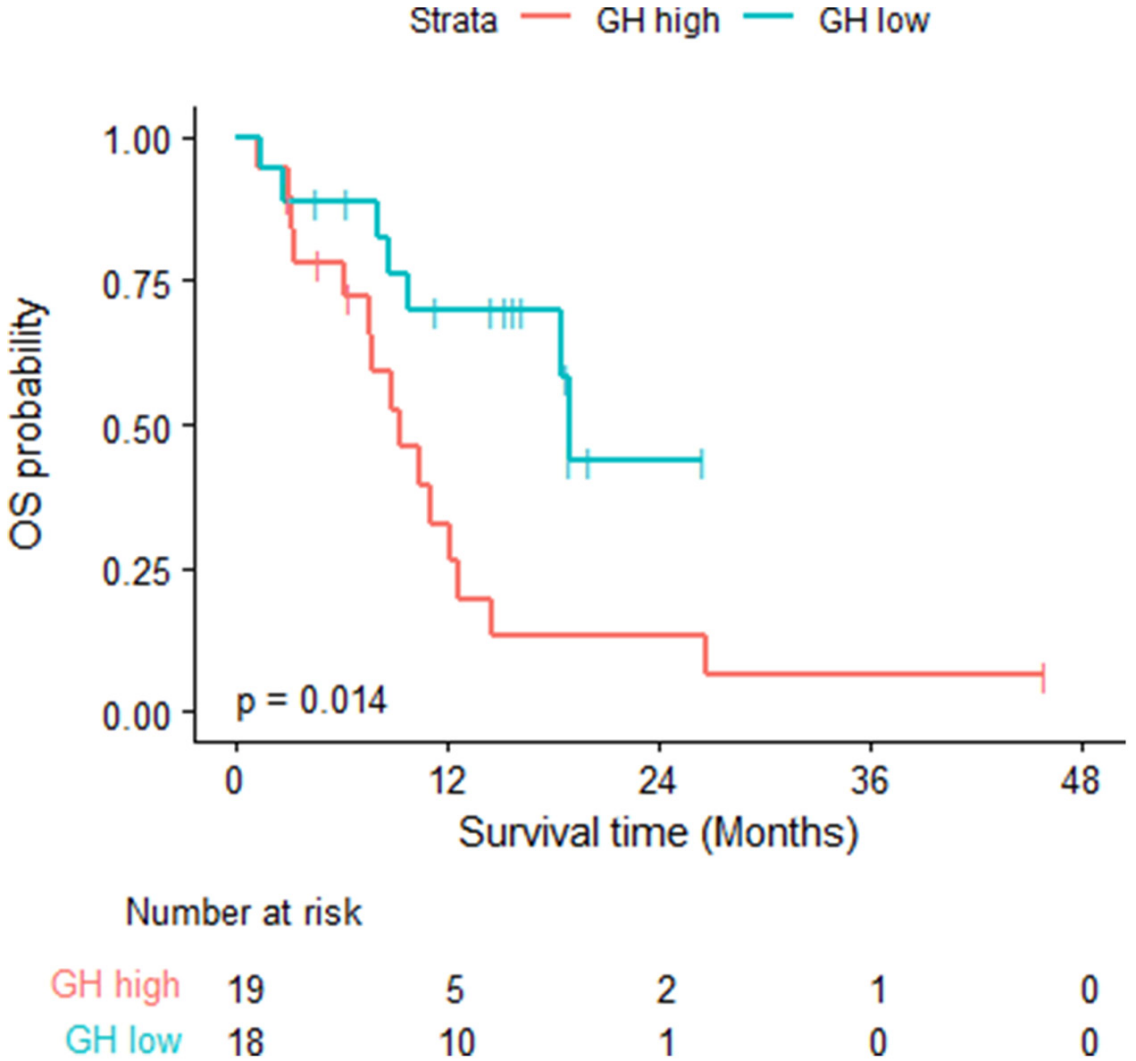
Overall survival- log rank test was used; fifteen of the 19 GH-high patients died, the median OS was 9.26 months (95% CI: 7.59, 14.59). Seven of the 18 GH-low patients died, median OS was 18.92 months (95% CI: 18.46, NA) (log rank test *p* = 0.0141).

GH levels for all patients were measured. At the time of the analysis, 22 of the 37 patients died, and the estimated median OS time was 12.16 months (95% CI: 9.26, NA). The estimated 1-year OS probability was 52% (95% CI: 0.37, 0.72). The median follow-up time was 18.8 months (95% CI: 15.3, NA). Fifteen of the 19 GH-high patients died, the median OS was 9.26 months (95% CI: 7.59, 14.59). Seven of the 18 GH-low patients died, median OS was 18.92 months (95% CI: 18.46, NA) (log rank test *p* = 0.0141). Table 2. Figure 1.

**Table 2:** Comparison of OS between GH-high and GH-low patients

	*N*	Event	Median OS (95% CI) (months)	1-year OS Rate (95% CI)	2-year OS Rate (95% CI)	*p*-value
	All patients	37	12.16 (9.26, N/A)	52% (37–72)	30% (16–55)	
**High**	19	15	9.26 (7.59–14.59)	33% (16–67)	13% (4–48)	0.0141
**Low**	18	7	18.92 (18.46-N/A)	70% (51–96)	44% (21–92)	

Abbreviations: GH: Growth Hormone; OS: overall survival; N/A: not applicable.

### Progression free survival (PFS)

**Figure 2:**
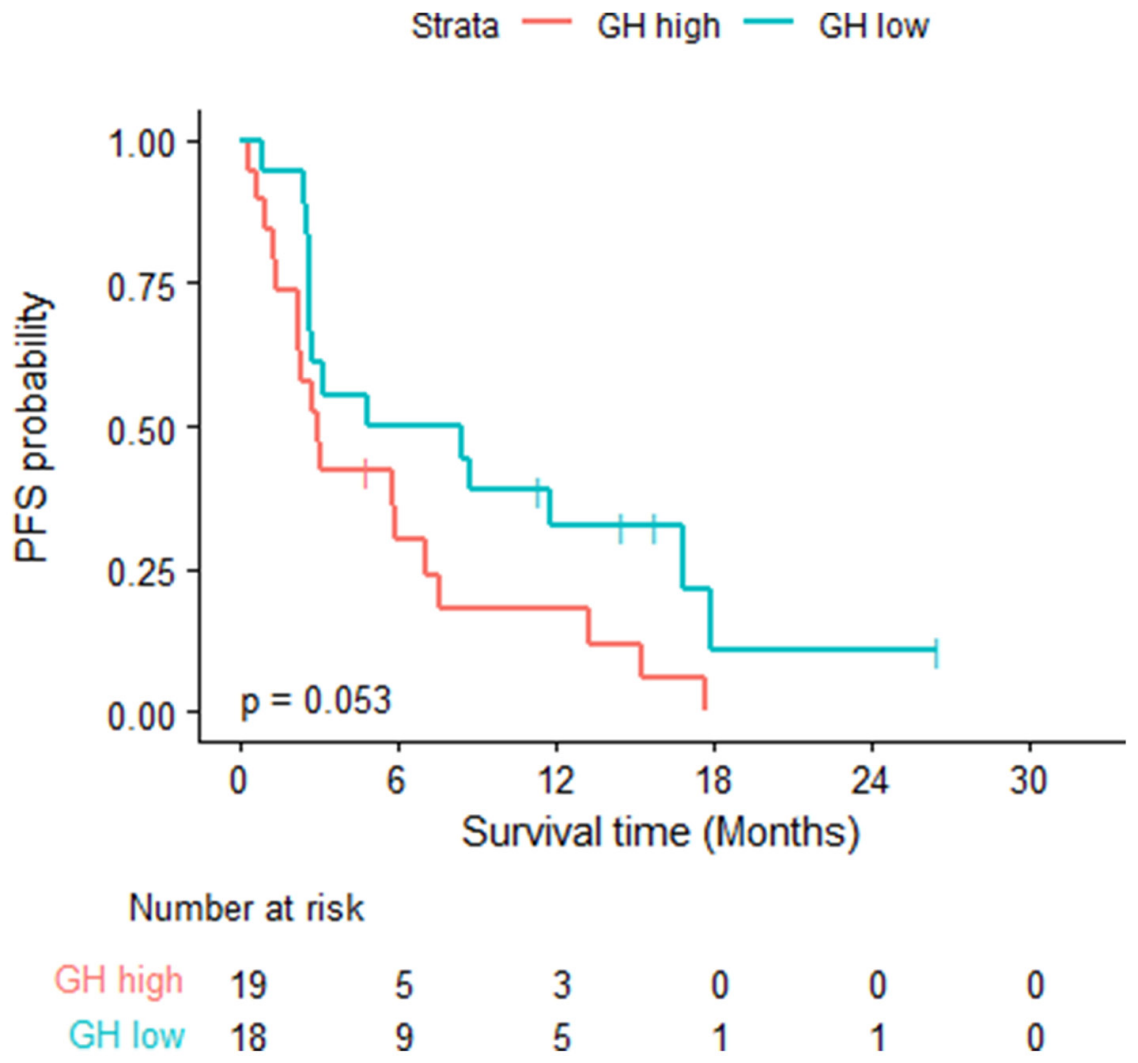
Progression free survival- log rank test was used; eighteen of the 19 GH-high patients had PD or died with a median PFS of 2.92 months (95% CI: 2.27, 7.56). 14 of the 18 GH-low patients had PD or died with a median PFS of 6.64 months (95% CI: 2.69, NA) (log rank test *p* = 0.0526).

Thirty-two of the 37 patients had PD or died, the estimated median PFS time was 3.12 months (95% CI: 2.69, 8.74). The estimated 6-month PFS probability was 40% (95% CI: 0.27, 0.6). Eighteen of the 19 GH-high patients had PD or died with a median PFS of 2.92 months (95% CI: 2.27, 7.56). Fourteen of the 18 GH-low patients had PD or died with a median PFS of 6.64 months (95% CI: 2.69, NA) (log rank test *p* = 0.0526). Table 3. Figure 2.

**Table 3:** Comparison of PFS between GH-high and GH-low patients

Plasma GH level	*N*	Event	Median PFS (95% CI) (months)	6-month PFS (95% CI)	*p*-value
	All patients	37	3.12 (2.69, 8.74)	40% (27, 60)	
**High**	19	18	2.92 (2.27–7.56)	30% (15–61)	0.0526
**Low**	18	14	6.64 (2.69-N/A)	50% (32–79)	

Abbreviations: GH: Growth Hormone; PFS: Progression-free survival; N/A: not applicable.

## DISCUSSION

In this study, we introduced a novel serum candidate biomarker, which is relatively cheap and minimally-invasive that can serve as a biological supplement to the already available prognostic factors to guide the selection of patients for treatment. Despite the small data size, plasma GH levels were strongly predictive of the disease outcome in patients treated with Atezo/Bev. This observation warrants validation studies which may eventually suggest a change in patient selection who could benefit from targeting GH/GHR signaling pathway and may provide an important prognostic biomarker for the selected patients.

Atezo/Bev regimen emerged as the preferred front-line treatment for advanced HCC as demonstrated by the IMbrave150 clinical trial [5], which enrolled advanced HCC patients to receive either the combination or sorafenib only. Atezo/Bev was tolerable and reported to be associated with some adverse events such as bleeding, hypertension, and proteinuria episodes, while sorafenib had more gastrointestinal and skin toxicities [5, 6]. Thus, choosing the right treatment for advanced HCC patient has to be weighed for risk-benefit ratio, in addition to comorbidities and tumor burden.

Systemic therapy in HCC carries a high cost, which adds a financial burden to the patients and healthcare systems [11]. Thus, it is important to know which patients will benefit from atezolizumab plus bevacizumab versus those in whom this combination will have lower survival benefit.

Despite this recent breakthrough in the treatment of patients with advanced HCC, there remains an unmet need for reliable biomarkers of response to immunotherapy.GH level have been reported and validated as a prognostic marker in HCC patients [7, 12, 13]. Our group reported in previous studies the prognostic value of circulating GH level in all 767 patients with different disease stages and treatment modalities received and also compared them to healthy volunteers (200 cases) [2, 13]. Our group also recently reported a significant correlation between GH level and overall survival [12]. Higher plasma GH levels were significantly correlated with thrombosis (*p* = .004), vascular invasion (*p* < .001) and tumor involvement of >50% liver (*p* = .003) and more advanced BCLC (*p* < .001) and TNM staging (*p* < .001). Median overall survival of patients without cirrhosis with GH-high levels was 13.1 months vs. 37.4 months for patients with plasma GH-low levels (*p* < .001).

In this study, we demonstrated the immediate clinical utility of the plasma GH level in patients treated with Atezo/Bev. Plasma GH level were independent prognostic factors in patients with advanced HCC patients who received atezolizumab plus bevacizumab. Revealing the prognostic value of plasma GH level for patients with advanced HCC in real-world clinical practice. Our study is the first to report GH level association with outcomes among patients with HCC treated with Atezo/Bev.

Patients with GH-high levels had significantly worse OS compared to those with lower level (9.27 months vs. 18.92 months) (*P* = 0.014). Patients with GH-high level had worse PFS compared to those with lower levels of plasma GH (2.92 months vs. 6.64 months) (*P* = 0.053). These results provides evidence of the clinical benefit of plasma GH as a biomarker for prediction and prognosis of patients with advanced HCC treated with Atezo/Bev.

Our study has some limitations. First, this investigation was conducted at a single center study in a selected population. Second, we have a small sample size of patients who have received the combination atezolizumab plus bevacizumab, which led to insufficient power to reach statistical significance for PFS, however, it showed a non-significant trend in favor of patients with plasma GH-low levels versus those with GH-high levels. A larger sample size with enough power will be required to validate these observations and confirm our results. Also, GH level has been reported to be associated with other systemic diseases, and therefore, future prospective studies are essential to study any interaction between different comorbidities and GH level in HCC patients and their potential effect on outcome.

To the best of our knowledge, this is the first study to assess clinical prognostic value of plasma GH level in patients who have received atezolizumab plus bevacizumab in clinical setting. Thus, the results of this study provide evidence to suggest the clinical utility of plasma GH as a potential noninvasive biomarker for prediction of OS and PFS in patients with HCC treated with Atezo/Bev. More importantly, it provides the basis to investigate the potential integration of anti-GH strategies, such as pegvisomant; a short peptide that inhibits the GH/GHR signaling and is the only drug approved by FDA to treat acromegaly [14], into systemic therapy approaches in HCC. Our group recently reported that GHR signaling could be a potential successful target in HCC [13].

Although this study focused on its baseline prognostic value, serial plasma GH level evaluation in future studies may also provide useful data to eventually guide therapy decision in clinical routine practice.

In conclusion, our study demonstrate that plasma GH represents a candidate biomarker for predicting treatment outcomes in patients with advanced HCC treated with Atezo/Bev. Future studies in larger randomized clinical trial and with a more diverse ethnic, race, and gender background are warranted to further validate these findings.

## MATERIALS AND METHODS

The University of Texas MD Anderson Cancer Center’s Institutional Review Board approved this study, and informed consent was obtained from all enrolled patients. We prospectively collected and measured pretreatment plasma GH levels of HCC patients who were treated with atezo/bev and followed up until progression and/or death and analyze correlation with pretreatment GH level. Patients received atezolizumab at a dose of 1200 mg and bevacizumab at a dose of 15 mg/kg intravenously every 3 weeks. Treatment continued until disease progression or the development of intolerable adverse events (AEs).

Adult patients with pathologically or radiologically confirmed HCC, as defined by the American Association for the Study of Liver Diseases, who were treated at MD Anderson Cancer Center (MDACC) from June 2018 to November 2021 and had pretreatment plasma GH level available were included in the study. Patients’ blood samples and epidemiologic and clinical data were collected, and plasma samples were analyzed retrospectively for GH level. Plasma GH levels were measured using an enzyme-linked immunosorbent assay (ELISA) and were used to stratify the HCC patients into high and low GH values (GH-high cutoff for women, >3.7 μg/L; men, >0.9 μg/L). Clinical and epidemiological data were retrieved from medical records. PFS was calculated from the date that Atezo/Bev treatment began to the date of disease progression or death, whichever occurred first. OS was calculated from the date that Atezo/Bev treatment began to the date of death or to the date of the last follow-up visit. The Kaplan-Meier method was used to calculate the time to event outcomes (i.e., OS and PFS) with Log rank test to compare OS or PFS between subgroups. This study was approved by MD Anderson Cancer Center’s Institutional Review Board.

## Abbreviations

CTP: Child-Turcotte-Pugh
ECOG: Eastern Cooperative Oncology Group
GH: Growth hormone
HCC: Hepatocellular carcinoma
HR: Hazard ratio
N/A: Not applicable
OS: Overall survival
PFS: Progression-free survival
